# Survey of abdominal obesities in an adult urban population of Kinshasa, Democratic Republic of Congo

**Published:** 2007-07

**Authors:** JB Kasiam Lasi On’kin, B Longo-Mbenza, A Nge Okwe, N Kangola Kabangu

**Affiliations:** Department of Internal Medicine, University of Kinshasa, Kinshasa, Democratic Republic of Congo; Department of Internal Medicine, University of Kinshasa, Kinshasa, Democratic Republic of Congo; Biostatistics Unit, Lomo Medical Centre and Heart of Africa Centre of Cardiology, Kinshasa, Democratic Republic of Congo; Biostatistics Unit, Lomo Medical Centre and Heart of Africa Centre of Cardiology, Kinshasa, Democratic Republic of Congo

## Abstract

**Background:**

The prevalence of overweight/obesity, which is an important cardiovascular risk factor, is rapidly increasing worldwide. Abdominal obesity, a fundamental component of the metabolic syndrome, is not defined by appropriate cutoff points for sub-Saharan Africa.

**Objective:**

To provide baseline and reference data on the anthropometry/body composition and the prevalence rates of obesity types and levels in the adult urban population of Kinshasa, DRC, Central Africa.

**Methods:**

During this cross-sectional study carried out within a random sample of adults in Kinshasa town, body mass index, waist circumference and fatty mass were measured using standard methods. Their reference and local thresholds (cut-off points) were compared with those of WHO, NCEP and IFD to define the types and levels of obesity in the population.

**Results:**

From this sample of 11 511 subjects (5 676 men and 5 835 women), the men presented with similar body mass index and fatty mass values to those of the women, but higher waist measurements. The international thresholds overestimated the prevalence of denutrition, but underscored that of general and abdominal obesity. The two types of obesity were more prevalent among women than men when using both international and local thresholds. Body mass index was negatively associated with age; but abdominal obesity was more frequent before 20 years of age and between 40 and 60 years old. Local thresholds of body mass index (≥ 23, ≥ 27 and ≥ 30 kg/m^2^) and waist measurement (≥ 80, ≥ 90 and ≥ 94 cm) defined epidemic rates of overweight/general obesity (52%) and abdominal obesity (40.9%). The threshold of waist circumference ≥ 94 cm (90th percentile) corresponding to the threshold of the body mass index ≥ 30 kg/m^2^ (90th percentile) was proposed as the specific threshold of definition of the metabolic syndrome, without reference to gender, for the cities of sub-Saharan Africa.

**Conclusion:**

Further studies are required to define the optimal threshold of waist circumference in rural settings. The present local cut-off points of body mass index and waist circumference could be appropriate for the identification of Africans at risk of obesity-related disorders, and indicate the need to implement interventions to reverse increasing levels of obesity.

## Summary

According to experts at the World Health Organisation (WHO), general obesity-related, cardiometabolic and cancerous morbidity and mortality are major health problems worldwide.^1^-^10^ The obesity−diabetes mellitus epidemic (diabesity) clusters with other bioclinical disorders in defining the metabolic syndrome according to ethnicity.^3^,^4^ Because of globalisation, the obesity epidemic is no longer an issue of only developed countries.^8^ Indeed, WHO reports one billion and 300 thousand overweight and clinically obese subjects, respectively, throughout the world.^10^ The obesity epidemic is therefore extending to developing countries.^9^,^10^

However, in developing countries of sub-Saharan Africa such as the Democratic Republic of Congo (DRC), it is to some extent difficult to define obesity. The sub-Saharan African socio-cultural context and the HIV/AIDS epidemic favour a misconception about abdominal obesity, as it is considered a social achievement,^11^ and lack of HIV disease (stigmatisation). The present data on general obesity is based on the body mass index (BMI) > 28 kg/m^2^ cut-off point.^12^ It is therefore urgent to define overall and abdominal obesities using relevant and specific reference values^3^ in central Africans with increasing urbanisation, acculturation, westernisation, epidemiological, demographic and nutrition transitions.^8^,^13^-^17^ The consequence of these transforming processes is lifestyle changes, including physical inactivity and excessive intakes of saturated fats, alcohol and refined sugar.^14^,^18^ The use of relevant values of reference^19^ and international cut-off points of waist circumference (WC) and BMI^20^-^22^ might render an easier definition of the metabolic syndrome in Africans compared to other populations of the world.

This study sought to provide baseline and reference data on nutritional status and to determine prevalence of overall and abdominal obesities in sub-Saharan Africa, in comparison with international cut-off points, gender, age and cardiometabolic risk factors. As nutritional assessments need to be updated frequently, we also evaluated the trend of overall obesity in comparison with earlier reports.^12^

## Materials and methods

This cross-sectional study involved 11 511 apparently healthy adult Africans (15 years and older) randomly selected from five geographic sites of Kinshasa (eastern, western, northern, southern and central parts), the capital of DRC, with seven million inhabitants. Selection was done by multi-stage sampling. In each geographic site, a number of quarters was randomly selected according to the population size (unpublished data of Kinshasa town): Pascal, Saint Theresa Square and Kingasani ya suka for the eastern part; Rond Point Victoire Square for the central part; Kintambo Magasin and Rond Point Kinsuka for the part; Rond Point Ngaba and Kinshasa University campus for the southern part. In the selected quarters, one street was then selected and adults of randomly selected households were invited to participate in the study.

Permission for the study was obtained from the supervising department of Kinshasa. In each quarter, the local administration gave its consent and explained the nature of the study to all residents. All the participants recruited gave their verbal consent according to the Helsinki Declaration II. The study was approved by the research and ethics committee of Kinshasa University.

Data were collected from 2 to 29 September 2002. Gender and ages were filled in by the principal investigator (KLJB) according to identity cards. Body weight was recorded to the nearest 0.1 kg using an electronic beam balance scale, with the participants wearing light indoor clothing and no shoes. Height was measured to the nearest 1 mm using a standardised wall-mounted height board. Waist circumference (WC) was measured to the nearest 1 mm at the level of the umbilicus and the superior iliac crest, at the end of normal expiration, using a non-extensible and flexible tape on subjects in a standing position. Measurements were made on a flat surface, with the weighing scale regularly calibrated before use and securely positioned on the floor. Body mass index (BMI) was calculated as the body weight (kg) divided by the height squared (m2).

## International cut-off points of nutritional status

The cut-off points of BMI established by WHO21 defined denutrition/underweight (< 18.5 kg/m^2^), normal nutritional status (18.5–24.9 kg/m^2^), overall overweight (25–29.9 kg/m^2^), and overall obesity (≥ 30 kg/m^2^). The degree of overall obesity was also quantified: grade (rank) I (30–34.9 kg/m^2^) grade II (35–39.9 kg/m^2^), grade III (≥ 40 kg/m^2^).

Abdominal (visceral) obesity was defined according to ATP III thresholds (≥ 102 cm for men and ≥ 88 cm for women)^22^ and the International Diabetes Federation (IDF) for Europid and other multiracial cut-off points (≥ 94 cm for men and ≥ 80 cm for women) (available from http://www.idf.org/webdata/docs/Metabolic_syndrom_definition.pdf).

The body composition for each participant included the total body water (TBW) calculated by the regression equation of Mellits and Check^25^ from deuterium oxide measurements [TBW in litres = 210.313 1 0.252 (weight in kg) 1 0.154 (height in cm) when ≥ 110 cm]; the lean body mass (LBM) in kg was derived from the method of Pace and Rathbun^26^ [LBM in kg = 0.72 (weight in kg)], and the body fat mass (BFM = body weight in kg 2 LBM in kg).

## Sub-Saharan Africa-specific thresholds for anthropometry

The local percentiles, reference limits (2.5 and 97.5 percentiles), the 0.90 confidence interval (CI) of the reference limits (lower CI limit = percent limit 22.81 3 SD/√n for Gaussian distribution; lower CI limit = e^-0.573^ and upper CI limit = e^-0.483^ following logarithmic transformation for non-Gaussian distribution), the quartiles (QI−IV), and the tertiles (TI-III) of the nutritional/body composition data were calculated.

The disorders of the nutritional status/body composition were established for both men and women (similar values of weight and body fat mass) from the normal values (2.5–97.5th percentiles), the population-based reference values (reference limits), the interquartile range (25–75th percentiles), the 2.5–50th percentile range and the tertile I of the measurements. Thus, following local and appropriate cut-off points, we defined Africa-specific nutritional disorders as: underweight/denutrition (< 2.5th percentile of BMI, WC, BFM), normal nutritional status (the range between 2.5th and 75th percentile of BMI, WC and BFM), overall overweight (50−75th percentiles of BMI), overall obesity (≥ 75th percentile of BMI, grade I with 75−90th percentile of BMI, grade II or severe with 90−97.5th percentile of BMI, and grade III or very severe with > 97.5th percentile of BMI), abdominal overweight (50−60th percentiles of WC), and abdominal obesity (≥ tertile II of WC, grade I with 60−75th percentiles of WC, grade II with 75−90th percentiles of WC, and grade III with ≥ 90th percentile of WC).

## Clinical insulin resistance defined by WC ≥ 94 cm

The defined local levels of cardiometabolic risk (not precise: no risk, light, moderate, high, very high risk) according to the degree of nutritional status will be used in the future prediction of the components of sub-Saharan Africa-specific metabolic syndrome (diabetes mellitus, arterial hypertension).

## Statistical analysis

Statistical analyses were performed using the statistical package for social sciences (SPSS) version 10.0 on Windows, Excel and R software.^27^ Analyses were stratified by gender, year of study,^12^ types of thresholds and degree of nutritional disorder. For the purpose of comparisons, the Chi-square test for percentages, Students *t*-test and one-way ANOVA test for means were used. The Scheffe *post hoc* test was used to determine significant differences. The simple correlation coefficient *r* was calculated between age and BMI. A *p*-value < 0.05 was considered statistically significant.

## Results

**Fig. 1.**
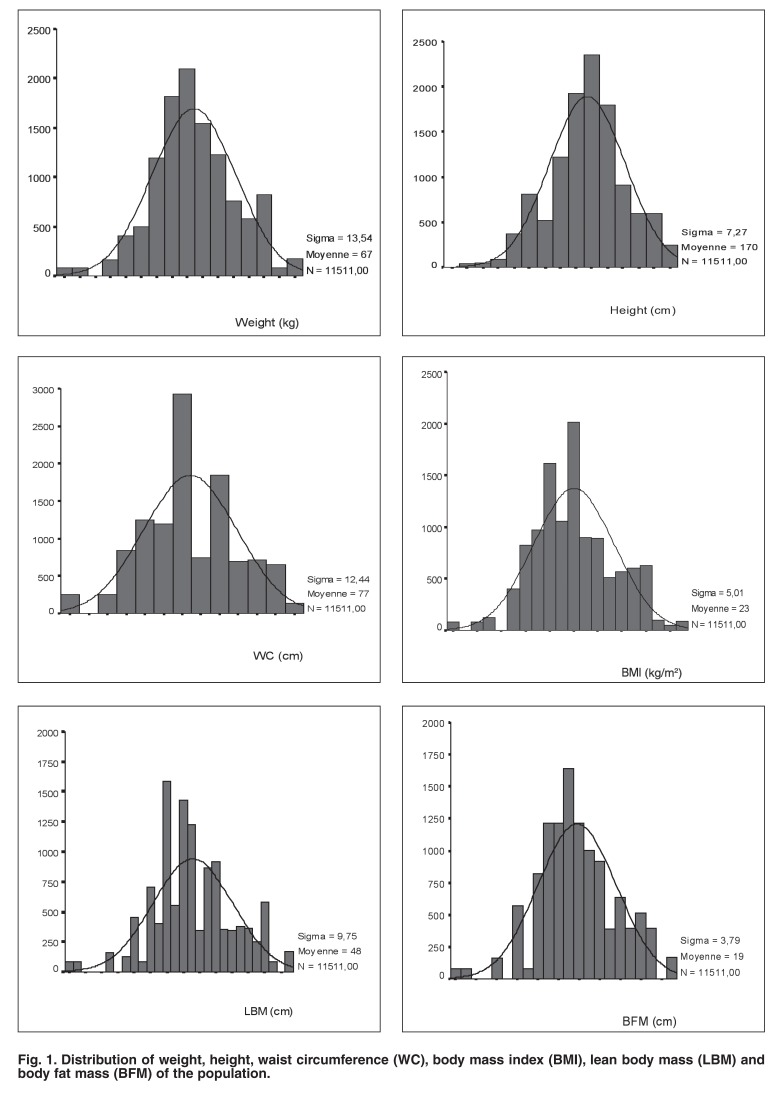
Distribution of weight, height, waist circumference (WC), body mass index (BMI), lean body mass (LBM) and body fat mass (BFM) of the population.

A total of 11 511 participants (response rate of 100%, 5 676 men, 5 835 women, mean age 37 ± 16 years) attended the morning mobile examination centre. The distribution of the nutritional status/body composition was Gaussian (Fig. 1). Table 1 presents the ranges, the normal values, means, percentiles, quartiles and reference limits of BMI, WC and BFM in the population.

**Table 1:** Local Characteristics Of The Study Population

*Local values*	*BMI (kg/m^2^)*	*WC (cm)*	*BFM (kg)*
Range	9–37	46–104	46–104
Quartile I	9–20	46–68	7–16
Quartile II	20−23	68–76	16–18.5
50−60th percentiles	20–23.9	76–79.9	18–18.9
60−70th percentiles	24–24.9	80–83.9	19–19.9
70−80th percentiles	25–27.9	84–86.9	20–21.9
Quartile III or 75th percentile	23–26	76–85	18.5–21
80−90th percentiles	28–29.9	87–93.9	22–23.9
≥ 90th percentile	> 30	> 94	> 24
Mean ± SD	23 ± 5	77 ± 12	19 ± 4
Normal values	15–33	54–100	11–26
References limits	15−33	54−100	11–26

The WHO cut-off points overestimated the prevalence rates of general denutrition, but underestimated those of overall over weight and obesity (52%) in comparison with the respective rates calculated by the local thresholds (Table 2).

**Table 2:** Prevalence Rates Of Nutritional Disorders Defined By The WHO And Local Cut-Off Points Of BMI

*Nutritional disorders*	*WHO BMI cut-off points n (%)*	*Local BMI cut-off points n (%)*
Denutrition	1852 (16.1)	288 (2.6)
Normal weight	5867 (51)	4967 (45.3)
Overall overweight	2327 (20.2)	3169 (28.9)
Overall obesity	1465 (12.7)	2547 (23.2)
Grade I	1329 (11.5)	738 (6.4)
Grade II	136 (1.2)	1231 (10.7)
Grade III	0 (0)	136 (1.2)

WHO: World Health Organisation.

Despite the younger age of the female participants, mean values of the nutritional status/body composition data of the women were similar to those of the men (Table 3). The overall obesity rate defined by the WHO criteria (BMI ≥ 30 kg/m^2^) in men (12.2%, *n* = 691) was similar (*p* = 0.083) to that of women (13.3%, *n* = 774). However, the overall obesity rate estimated by the local threshold (BMI ≥ 27 kg/m^2^) was higher (*p* < 0.0001) in women (24.4%, *n* = 1 422) than in men (19.8%, *n* = 1 125).

**Table 3:** Comparison Of Characteristics Of Men With Those Of Women

*Variables*	*Men*	*Women*	p*-value*
Age (years)	37 ± 16	36 ± 16	< 0.001
BMI (kg/m^2^)	23.4 ± 4.7	23.6 ± 5.3	NS
WC (cm)	77.4 ± 12.3	77 ± 12.6	NS
Lean body mass (kg)	48.5 ± 8.7	48.2 ± 10.7	0.143
Body fat mass (kg)	19 ± 4	19 ± 4	0.143
Total body water (l)	33 ± 3	33 ± 3	0.517

NS: *p* > 0.05

Table 4 presents the rates of abdominal obesity according to international and local thresholds. The rates of abdominal obesity estimated by the IDF criteria in men and women were similar (*p* > 0.05) to those defined by local criteria (without difference between men and women), respectively. However, the ATP III criteria underestimated the rates of abdominal obesity in men as well as in women.

**Table 4:** Prevalence Rates Of Abdominal Obesity Estimated By International And Local Cut-Off Points

*Gender*	*ATP III cut-off points n (%)*	*IDF cut-off points n (%)*	*Local cut-off points n (%)*
Men	133 (2.3) by	319 (5.6) by	319 (5.6) by
	WC ≥ 102 cm	WC ≥ 94 cm	WC ≥ 94 cm
			2385 (40.9) by
			WC ≥ 80 cm
Women	954 (16.3) 2385 (40.9)	2385 (40.9) by	2385 (40.9) by
	by WC ≥ 88 cm	WC ≥ 80 cm	WC ≥ 80 cm
			319 (5.6) by
			WC ≥ 94 cm

WC: waist circumference.

**Fig. 2.**
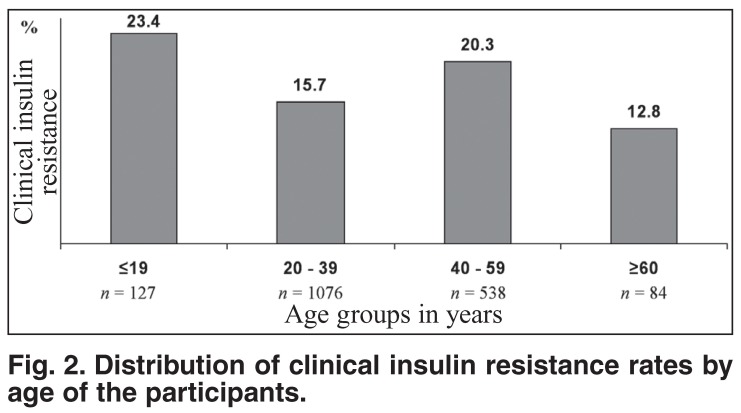
Distribution of clinical insulin resistance rates by age of the participants.

**Fig. 3.**
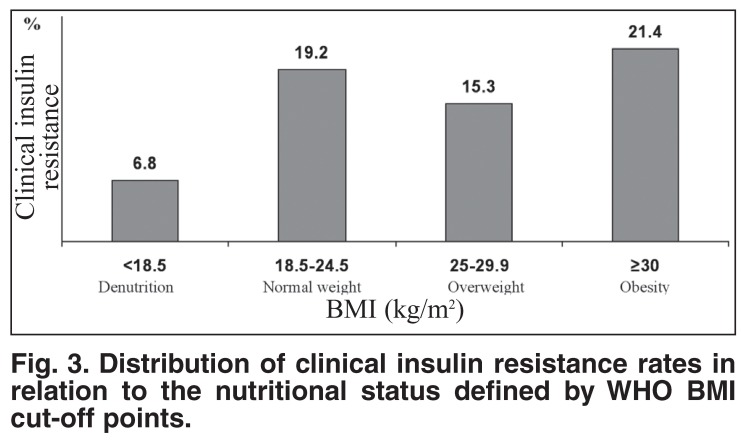
Distribution of clinical insulin resistance rates in relation to the nutritional status defined by WHO BMI cut-off points.

**Fig. 4.**
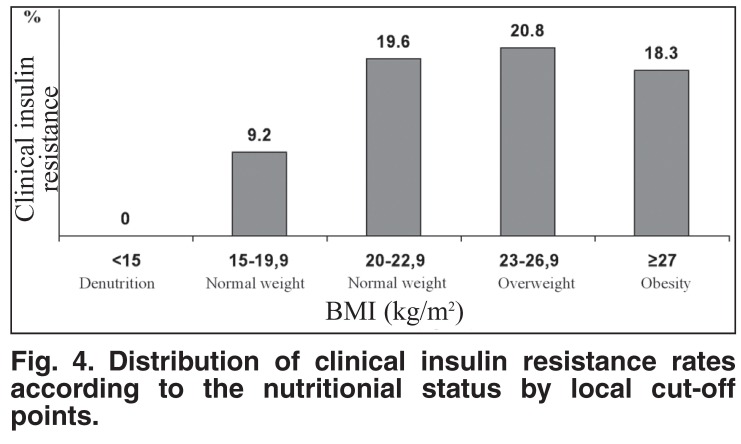
Distribution of clinical insulin resistance rates according to the nutritionial status by local cut-off points.

Despite the existing inverse relationship between clinical insulin resistance and age of participants, the highest rates of clinical insulin resistance were present among participants aged 19 years or younger and between 40 and 59 years (Fig. 2). The distribution of clinical insulin resistance rates by the degree of overall obesity defined by WHO criteria showed a paradoxical presence of clinical insulin resistance among participants with overall denutrition, and higher rates of clinical insulin resistance in participants with normal weight, in comparison with those who were overweight (Fig. 3). However, clinical insulin resistance was not present in denutrition and increased with the degree (positive relationship) of overall obesity defined by local BMI cut-off points (Fig. 4).

**Fig. 5.**
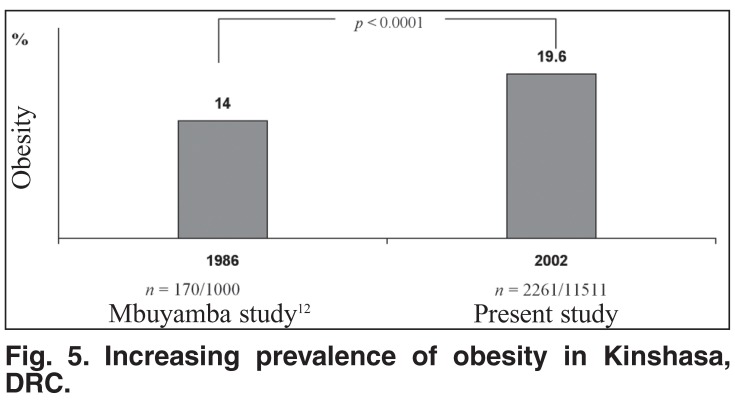
Increasing prevalence of obesity in Kinshasa, DRC.

Using BMI ≥ 28 kg/m^2^ as the cut-off point, the present rate of obesity was higher (*p* < 0.0001) than that reported in 198612 and suggested an increasing trend of obesity (Fig. 5). Age was negatively correlated (*r* = 20.030; *p* < 0.0001) with BMI values.

Finally, Table 5 provides a definition of the cardiometabolic risk according to sub-Saharan Africa-specific levels of nutritional disorders quantified by different levels of BMI, WC and BFM. This cardiometabolic risk profile will serve in future to identify individuals at higher risk for type 1 and 2 diabetes.

**Table 5:** Definition Of Levels Of Cardiometabolic Risk By Different Local Cut-Off Points Of BMI, WC And BFM

*Nutritional status*	*Local cut-off points of BMI*	*Local cut-off points of WC*	*Local cut-off points of BFM*	*Cardiometabolic risk*
Denutrition	< 15	< 54	< 11	Undetermined
Normal weight	15–22.9	54–75	11–18.9	Reference
Overweight	23–26.9	76–79	19–20.9	Light
Obesity				
Grade I	27–29.9	80–85	21–21.9	Moderate
Grade II	30–33.9	86 – 93	22–23.9	High
Grade III	≥ 34	≥ 94	≥ 24	Very high

BMI: body mass index, WC: waist circumference, BFM: body fat mass.

## Discussion

This is the first study in Kinshasa to assess indices and disorders of nutrition. The present data on anthropometry and body composition in central Africans offer significant contributions, not only in providing an understanding of the index of health and well-being at both the individual and population levels, but also in terms of cardiometabolic risk.

Until now, African studies were limited to estimating the prevalence of overall overweight/obesity using different cutoff points of BMI.^12^,^27^ Concern for a new worldwide definition of the metabolic syndrome^3^,^4^ has arisen from the lack of sub-Saharan Africa-specific cut-off points of waist circumference. Therefore, the need to identify the status of overall and abdominal overweight and obesity among African adults is important so that effective intervention programmes can be implemented at an early stage.

The first objective of this study was to establish local normal and reference values of body mass index, waist circumference and body fat mass at the population level. The normal values were roughly equivalent to the reference values of each nutritional parameter.

Using standardised methods, international thresholds,^19^-^21^ and local criteria (the cut-off points for overall overweight and overall obesity would have to be about 23−26.9 kg/m^2^, respectively; the cut-off points for abdominal obesity/clinical insulin resistance ≥ 80 cm, ≥ 90 cm and ≥ 94 cm; excess of body fat mass ≥ 21 kg), the present study reports higher rates of overweight/obesity in comparison with previous data from the same background.^12^,^29^ The lifestyle changes related to urban migration, industrialisation, westernisation,^13^ epidemiological and nutrition transitions^16^,^17^ may explain the difference between developing countries and developed nations.^8^-^10^

As reported for industrialised countries,^2^-^6^,^28^ the present high rates of overall and abdominal obesities might also explain the emergence of non-communicable diseases (arterial hypertension, dyslipidaemia, diabetes mellitus, cancers and cardiovascular diseases) in sub-Saharan Africa.^30^-^33^ However, the WHO criteria^23^,^24^ tended to underestimate the prevalence of overall overweight and overall obesity, and to overestimate the prevalence of denutrition in comparison with local criteria. The prevalence of abdominal obesity in men and women, respectively, was underestimated by the ATP III cut-off points.^21^,^22^ The underestimation of overall and abdominal obesities in central Africans was also reported in Cameroon.^29^ Curiously, the IDF thresholds to define abdominal obesity (central adiposity) rates for Europid, sub-Saharan African, Eastern and Middle-Eastern populations (WC ≥ 94 cm for men and ≥ 80 cm for women) underestimated rates of abdominal obesity in comparison with local cut-off points for WC.

In considering only waist circumference ≥ 94 cm to define clinical insulin resistance, the WC ≥ 94-cm cut-off point (≥ 90th percentile of WC) corresponded to body mass index ≥ 30 kg/m^2^ (≥ 90th percentile of BMI) and body fat mass ≥ 24 kg (90th percentile of BFM). Therefore, WC ≥ 94 cm defined individuals with a very high cardiometabolic risk in this African population. WC ≥ 94 cm and ≥ 80 cm for men and women, respectively provided the same rates of abdominal fat as reported in Cameroon for WC ≥ 94 cm.^29^

The present study shows that clinical insulin resistance (WC ≥ 90 cm including grades II and III of abdominal obesity) was present in each category of nutritional disorder defined by WHO BMI criteria,^23^,^24^ including denutrition. The merit of the local BMI criteria to define nutritional disorders was the absence of clinical insulin resistance within the denutrition category. These results suggest that BMI cut-off points (mostly BMI < 30 kg/m^2^) do not evaluate the same excess of fat mass^29^,^35^ and the fat-related cardiometabolic risk in comparison with WC cut-off points. Indeed, WC ≥ 94 cm compared with BMI ≥ 30 kg/m^2^ was the better predictor of arterial hypertension in a working population of Africans in Kinshasa.^36^ This is because the visceral fat distribution (abdominal obesity) is metabolically more active than the subcutaneous fat, and therefore more harmful for health in general,^37^ and risk of atherosclerotic diseases in particular.^38^,^39^

Therefore, waist circumference is the best simple anthropometric index of abdominal and visceral adipose tissue accumulation and is proposed as a surrogate of insulin resistance/hyperinsulinaemia in sub-Saharan Africa with low resources and low values of serum total cholesterol and triglycerides, but high levels of HDL cholesterol.^31^-^33^ As the measurement of waist circumference is recommended to identify individuals requiring intervention to reduce cardiometabolic risk,^40^ the present study proposes the use of the following categories to indicate in combined male and female Africans: low cardiometabolic risk, < 80 cm; increased risk, 80−93 cm; and substantially increased risk, ≥ 94 cm. These cut-offs correspond to BMI of < 27 kg/m^2^, 27−30 kg/m^2^, and ≥ 30 kg/m^2^, respectively.

The concomitant presence of denutrition and obesity means that these young adults (37 ± 16 years) are facing epidemiological, demographic and nutrition transitions.^8^,^14^-^16^ Furthermore, the economy of DRC has been in recession with predictably deleterious effects on these participants with denutrition: a decrease in body weight and body fat associated with inadequate food and energy supplies. However, some Africans with denutrition and higher levels of WC will have a higher risk of arterial hypertension, as demonstrated among African children from Kinshasa with higher blood pressure and heart rate in comparison with their normal-weighted counterparts.^41^

The progression to the metabolic syndrome and atherosclerotic diseases for Africans with denutrition and normal weight may be explained by the positive relationship between *Helicobacter pylori* and higher waist circumference and blood pressure, higher levels of total cholesterol and fibrinogen, but lower HDL cholesterol levels than shown in lean adult Africans.^42^

Contrary to Africans from Cameroon with a positive relationship between age and BMI levels,^29^,^43^ the Africans in this study from Kinshasa showed a negative correlation between age and BMI levels. Furthermore, despite the highest rates of clinical insulin resistance (WC ≥ 90 cm) observed for ages 19 years and younger and between 40 and 59 years, globally there was a negative correlation between clinical insulin resistance and age of participants.

We understand now why the age of 60 years or older is one of the risk factors of the epidemic of ischaemic stroke among Congoleses patients,^32^,^33^ as individuals from 40 to 59 years have a higher cardiometabolic risk. The present findings also suggest that the risk of the metabolic syndrome (type 2 diabetes mellitus and arterial hypertension) would appear before the age of 20 years and after the age of 40 years. This observation explains the difficulty we are facing to classify diabetes mellitus and to define type 2 diabetes in Kinshasa.

## Conclusion

There were differences in defining prevalences of overall and abdominal obesities when WHO criteria, ATP III and local Africa-specific cut-off points of BMI and WC were used; transient IDF thresholds for sub-Saharan Africa were equivalent to local Africa-specific cut-off points of WC. As WHO criteria and ATP III cut-off points underestimated the prevalence of overall overweight/obesity and abdominal obesity, it was necessary to take into account age, body shape, nutrition transition, economic recession and ethnicity before interpreting BMI and WC in terms of cardiometabolic risk.

Not taking into account gender for sub-Saharan Africa, the following cut-offs are recommended to identify individuals at higher cardiometabolic risk: low risk (BMI < 27 kg/m^2^ and WC < 80 cm), increased risk (BMI 27−30 kg/m^2^ and WC 80−93 cm), and substantially increased risk (BMI ≥ 30kg/m^2^ and WC ≥ 94 cm).

Increasing and epidemic levels of overall overweight/obesity and abdominal obesity (52%) has caused the need for further serial urban and rural studies to monitor trends and validate optimal thresholds to establish a new worldwide definition of the metabolic syndrome.
